# Ventricular cell fate can be specified until the onset of myocardial differentiation

**DOI:** 10.1016/j.mod.2016.01.001

**Published:** 2016-02

**Authors:** Simona Caporilli, Branko V. Latinkic

**Affiliations:** School of Biosciences, Cardiff University, Museum Avenue, Cardiff CF10 3AX, Wales, UK

## Abstract

The mechanisms that govern specification of various cell types that constitute vertebrate heart are not fully understood. Whilst most studies of heart development have utilised the mouse embryo, we have used an alternative model, embryos of the frog *Xenopus laevis*, which permits direct experimental manipulation of a non-essential heart. We show that in this model pluripotent animal cap explants injected with cardiogenic factor GATA4 mRNA express pan-myocardial as well as ventricular and proepicardial markers. We found that cardiac cell fate diversification, as assessed by ventricular and proepicardial markers, critically depends on tissue integrity, as it is disrupted by dissociation but can be fully restored by inhibition of the BMP pathway and partially by Dkk-1. Ventricular and proepicardial cell fates can also be restored in reaggregated GATA4-expressing cells upon transplantation into a host embryo. The competence of the host embryo to induce ventricular and proepicardial markers gradually decreases with the age of the transplant and is lost by the onset of myocardial differentiation at the late tailbud stage (st. 28). The influence of the host on the transplant was not limited to diversification of cardiac cell fates, but also included induction of growth and rhythmic beating, resulting in generation of a secondary heart-like structure. Our results additionally show that efficient generation of secondary heart requires normal axial patterning of the host embryo. Furthermore, secondary hearts can be induced in a wide range of locations within the host, arguing that the host embryo provides a permissive environment for development of cardiac patterning, growth and physiological maturation. Our results have implications for a major goal of cardiac regenerative medicine, differentiation of ventricular myocardium.

## Highlights

• Ventricular and proepicardial fate can be induced by cardiogenic factor GATA4.
• This process requires tissue integrity.
• Ventricular and proepicardial cell fate can be restored by BMP inhibition.
• A secondary heart-like structure can be induced from GATA4-expressing cells.

## 1 Introduction

Heart is the first functional organ in developing vertebrate embryos. It forms through an overlapping sequence of specification, differentiation and morphogenesis steps. Much of what is known about heart development was derived from genetic analyses in the mouse. However, one limitation of the mouse model derives from the essential role of the heart during embryonic development as well as exquisite sensitivity of the heart to systemic perturbations. Complementary models for studying heart development that compensate for these limitations are zebrafish and *Xenopus* embryos, as their development until later stages does not require a functional heart.

*Xenopus* heart is composed of three chambers, one ventricle and two atria, and is evolutionarily located between the two-chambered fish heart and the four-chambered amniote heart (Warkman and Krieg, 2007). These morphological differences are not restrictive because heart development is governed by a core of evolutionally conserved transcription factors (Gessert and Kuhl, 2009, Olson, 2006). The conservation between *Xenopus* and mouse heart development extends to expression of cardiac transcription factors in First and Second Heart Fields (Gessert and Kuhl, 2009).

Heart mesoderm specification in *Xenopus* is thought to occur during gastrulation (Sater and Jacobson, 1989) and cardiac precursors subsequently migrate towards the anterior end, moving on laterally to fuse on the ventral midline to form a delta-shaped sheet from which a linear heart tube will be generated (Kolker et al., 2000). At this stage the heart tube expresses pan-myocardial markers myl7 (synonym MLC2; (Latinkic et al., 2004)), and myh6 (synonym MHCα (Logan and Mohun, 1993, Mohun et al., 2000)) and is located along the anteroventral region behind the cement gland along the embryonic A-P axis (Mohun et al., 2000). The expression of myl7 and myh6 indicate the onset of cardiac differentiation. At stage 35 the linear heart tube changes its anterior–posterior axis direction because the posterior side (presumptive atrium and sinus venous) moves dorsally, more closely to the anterior region and the heart tube moves anticlockwise in a spiral motion in the process known as heart looping. Consequently, the outflow tract (OFT, positioned anteriorly) moves medially and the conus (positioned posteriorly) relocates to the right side of the embryo. During heart chamber formation (stage 39–40) the atrium is located dorsally compared to the ventricle and at the same time the development of ventricular trabeculae and valves occurs.

The outer layer of the vertebrate heart, the epicardium, originates from the proepicardium which develops in the posterior limits of the heart between the sinus venosus and the liver (Manner et al., 2001). Mesenchymal cells delaminate from epicardium and invade the myocardium and subepicardial spaces to provide the cardiac vessels and cardiac fibroblasts. The epicardium is characterised by restricted (but not epicardial-specific) expression of transcription factors Wilm's tumour (WT-1), Pod-1/epicardin (Perez-Pomares et al., 2009, Ratajska et al., 2008) and Tbx18 (Cai et al., 2008, Jahr et al., 2008) and is involved in the proliferation of the embryonic myocardium (Manner et al., 2001, Perez-Pomares et al., 2009, Smith and Bader, 2007).

The chamber myocardium is generated from a pool of rapidly proliferating cardiac precursor cells. As the precursors differentiate into cardiomyocytes proliferation slows down but their size increases. There is the inverse relationship between the proliferation and differentiation: in *Xenopus*, cyclin-dependent kinase inhibitor p27Xic is required for cardiac differentiation (Movassagh and Philpott, 2008).

Regionalisation of the developing heart is manifested by the restricted expression of genes such as myl3, irx4 and nppa. myl3 (synonym MLC1v) is a regionalised myocardial marker that is expressed after heart looping (stage 36) in the ventricle; outside of the heart myl3 is dynamically expressed in somites and hyoid muscles (Smith et al., 2005). Iroquois 4 (irx4) homeodomain transcription factor is involved in ventricular development, and its expression in *Xenopus* becomes restricted to the ventricle within the heart after stage 39 (Garriock et al., 2001, Smith et al., 2005). Despite the number of genes known to be regionally expressed within the differentiated myocardium in *Xenopus*, still there are no specific atrial markers at the time when ventricular differentiation is ongoing. A well described atrial marker in all vertebrates, atrial natriuretic factor (nppa), is expressed throughout the developing myocardium from stage 32, and only at stage 49 its expression is exclusively seen in the atrium (Small and Krieg, 2000).

To investigate specification of cardiac cell types (diversification of cardiac fates) we used *Xenopus* pluripotent blastula stage animal cap explants. Expression of GATA4 in animal cap explants has been known to induce pan-myocardial cardiac differentiation marker myl7 and beating tissue (Latinkic et al., 2003), but specification of other cardiac fates has not been reported. Here we show, using GATA4 as a trigger of cardiogenesis in animal cap explants, that ventricular and proepicardial (VP) cell fates are induced in a process that requires tissue integrity. Practical limit for culturing animal cap explants is until around st. 40, well before nppa becomes restricted to the presumptive atria, and has precluded us from directly establishing atrial cardiomyocyte fate. Instead we have relied on indirect method afforded by double whole-mount in situ hybridisation (double WMISH) with a panmyocardial (e.g. myl7) and a ventricular marker such as myl3 and considering a myl7^+^/myl3^−^ area as atrial. In GATA4-expressing explants whose integrity has been disrupted by dissociation, VP fates can be rescued by inhibition of BMP signalling. Cultured animal cap explants, however, are not an optimal model to study cardiac morphogenesis and growth. To examine these aspects of heart development we used an alternative assay in which GATA4-injected dissociated animal cap explants transplanted into host embryo develop a secondary ‘heart’, characterised by cardiac cell diversification, growth, beating and limited morphogenesis. Using this assay we show that VP fates can be specified in GATA4-induced cardiac precursors by the signal(s) provided by the host embryo until the onset of cardiac differentiation.

## 2 Materials and methods

### 2.1 *Xenopus* embryos, microinjection and explants

*Xenopus* embryos were obtained, injected and cultured in 10% Normal Amphibian Medium (NAM; 110 mM NaCl, 2 mM KCl, 1 mM Ca(NO_3_)_2_, 1 mM MgSO_4_, 0.1 mM EDTA, 1 mM NaHCO_3_) (Sive et al., 2000). Animal pole explants were excised on agarose-coated dishes (1% agarose in dH_2_O) at stage 8.5–9 and cultured in 75% NAM until the indicated stage. For dissociation, animal cap explants were cut in 95% Ca^2 +^/Mg^2 +^-free saline medium (CMFM; 88 mM NaCl, 1 mM KCl, 2.4 mM NaHO_3_, 7.5 mM Tris–HCl pH 7.6) and 5% of 75%NAM. Twenty five explants were transferred to a 1.5 ml tube and dissociated by gentle agitation in CMFM, 0.1% Bovine Serum Albovine (BSA, Sigma). The dissociated cells were centrifuged at 1000 rpm for 1 min and CMFM, 0.1% BSA was replaced with CMFM, 0.5 mM CaCl_2_. The cells were left for 3 h at 19–21 °C. The reaggregated cells (reaggregates) were transferred to agarose-coated dishes containing 75%NAM and gentamicine (25 mg/ml, Sigma-Aldrich) and cultured at 19–21 °C. Dexamethasone (2 μ M; Sigma), dissolved in ethanol, was added at the stages indicated. Transgenic frog line Cardiac actin-GFP has been described (Latinkic et al., 2002). Heart field explants were excised from the embryos at stage 20 as previously described (Raffin et al., 2000). Dissociation of heart field explants was carried out using CMFM with 0.1% BSA and reaggregation was carried out by adding 0.5 mM CaCl2 to CMFM. Heart Field were cultured in 0.75 × NAM until required stage. Axis perturbation by UV exposure was performed as previously described (Sive et al., 2000). UV-treated embryos were scored using the dorsoanterior index (DAI) (Sive et al., 2000). Irradiated embryos with DAI lower than 2 were chosen for further experiments (Sive et al., 2000).

Synthetic capped RNA was synthesised as described (Sive et al., 2000). The amounts injected were: 1 ng *Gata4*-GR (Afouda et al., 2005, Latinkic et al., 2003), 1 ng *Dkk1* (Glinka et al., 1998) and 1 ng tBr (Graff et al., 1994). mRNAs were coinjected together with rhodamine–dextran (20 mg/ml) and dextran–biotin (25 mg/ml) as lineage tracer in a final concentration of 10%.

Dissociated and reaggregated explants were transplanted at stage 20 in the ventral region of *Xenopus* host embryos at the same stage. Before transplantation reaggregated explants were cut to 0.03 mm in size using a 10 mm graticule (Tonbridge, Kent, England) placed under the agarose dish as a guide. Transplanted embryos were cultured in 0.75 × NAM with gentamicine (25 mg/ml, Sigma-Aldrich). Green Fluorescent Protein (GFP) presence was detected using a Leica MZ16F Fluorescence Stereomicroscope with a GFP filter (GFP2: excitation 480/40 nm, emission 510 nm).

### 2.2 Whole mount in situ hybridization

Whole-mount in situ hybridization (WMISH) was carried out by the method described (Sive et al., 2000), using the digoxigenin or fluorescein (Roche)-labelled antisense riboprobes. Templates for synthesising myl3 (Smith et al., 2005), myl7 (Latinkic et al., 2004) and tnni3 (Drysdale et al., 1994) have been described. Template for tbx18 was generated by cloning a PCR product obtained with primers used for RT-PCR analysis (see below) into pGemTEasy (Promega); SalI was used for linearization and T7 polymerase to synthesise antisense probe.

### 2.3 Immunofluorescence

Immunofluorescence was carried out as described (Kolker et al., 2000), using mouse anti-chicken tropomyosin monoclonal primary antibody (CH1; Developmental Studies Hybridoma Bank) − 1:50 and Goat α-mouse Alexa Fluo 488 (Invitrogen) − 1:500 as secondary antibody. For confocal analysis, samples were dehydrated in methanol, and cleared with benzyl alcohol/benzyl benzoate (Fisher Scientific).

### 2.4 RT-PCR

Total RNA was isolated from samples using the acid guanidinium thiocyanate-phenol-chloroform method (Chomczynski and Sacchi, 1987). 20–25 animal cap explants, 3 reaggregates, 5 HF explants or 5 control embryos were used per sample, and cDNA synthesised using MMLV-RT (Invitrogen, UK) and random hexamers according to manufacturer's specifications. PCR was carried out using GoTaq polymerase (Promega, UK) with the following cycling conditions: 95 °C for 30 s, 58 °C for 30 s, 72 °C for 30 s. Primer sequences: irx4 (GenBank No. NM_001096735) F: tgcagctttgggtgtctatg, irx4 R: atggccagcatgatcttctc; myl3 (AY289206) F: tgggacagaatccaaccaat, R: tgaatggtgttcctgtgcat; myl7 (AY219706) F: tgtatcgaccaaaaccgtga, R: attggggtcacagcaaacat; odc1 (NM_001086698) F: gccattgtgaagactctctccatt, R: ttcgggtgattccttgccac; tbx18 (EB736397) F: tgtttccagccatgagagtg, R: gagagatggctccaaaatgc; wt1 (BC170513) F: atcagtgtctcagcgccttt, R: gagaggtctgttggctgagg.

## 3 Results

### 3.1 GATA4 induces cardiac cell diversification in intact animal cap explants

**Fig. 1:**
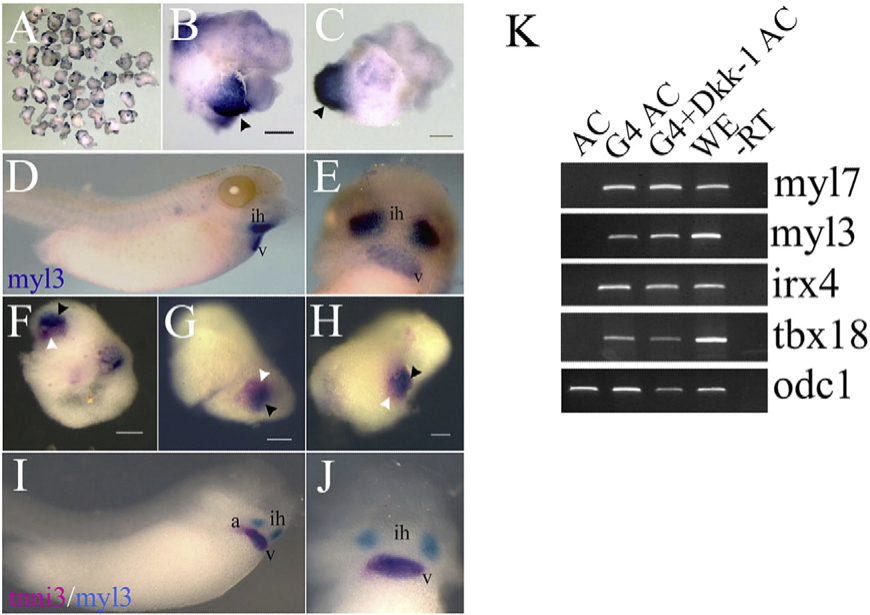
GATA4 induces the expression of ventricular and proepicardial markers in animal cap explants. (A) Ventricular gene expression is induced in animal cap explants injected with GATA4 (75%, n = 50) using WMISH analysis for the expression of myl3. (B, C) Close-up of animal cap explants shown in (A); black arrowheads are indicating myl3 expression. (D, E) Lateral and ventral views of a myl3 expression in control embryo (stage 39). (F–H) Animal caps injected with GATA4 showed incompletely overlapping expression of pan-myocardial marker tnni3 and ventricular marker myl3 (36% (n = 55), 13% showed tnni3 expression only). Similarly to the heart of control embryos (I, J), GATA4 injected AC explants show a region of overlapping expression (black arrowheads) and region expressing tnni3 alone (white arrowhead). (K) RT-PCR analysis in AC explants at stage 40 injected with GATA4 or co-injected with GATA4 and Dkk-1 mRNA showed the expression of the ventricular (myl3 and irx4) and proepicardial (tbx18) markers as in sibling control embryos at the same stage. A representative experiment out of 3 repeats is shown. a, atrium; ih, interhyoid muscle of the jaw; v, ventricle. The scale bar corresponds to 100 μm.

**Fig. 2:**
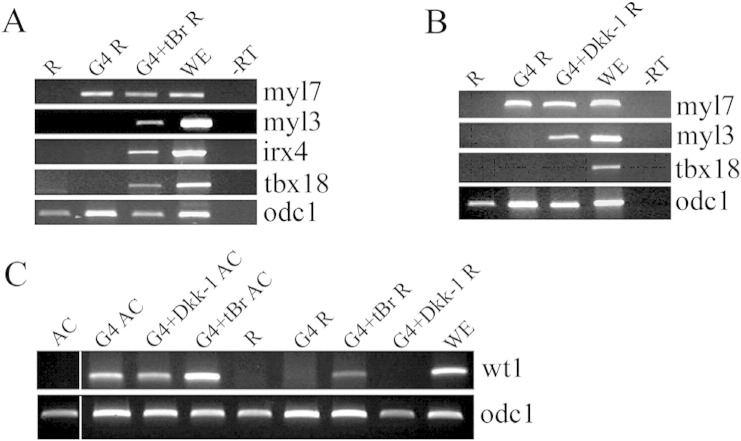
Cardiac cell diversification is lost in dissociated and reaggregated animal cap explants but can be restored by blocking BMP signalling. (A) Restricted cardiac gene expression is lost in GATA4 injected explants dissociated and reaggregated. Inhibition of BMP signalling through dominant-negative BMP type I receptor (tBr) restores ventricular and proepicardial gene expression in GATA4 co-injected reaggregated explants. (B). Ventricular gene expression (myl3) is partially restored in explants also co-injected with GATA4 and Dkk-1 mRNA. (C) The second proepicardial marker, WT1, shows the same pattern of expression as Tbx18 in intact (Fig. 1K) and reaggregated animal cap explants expressing GATA4, GATA4 and Dkk-1 or GATA4 and tBr. AC, Animal Caps; G4, GATA4; R, dissociated and reaggregated animal caps. RT-PCR was performed at stage 40 according to control embryos. A representative experiment is shown out of 3 (A, B) or 2 (C).

To study the mechanisms involved in cardiac cell fate diversification we used animal cap explants from blastula stage *Xenopus* embryos injected with GATA4 mRNA. Previous experiments showed that explants injected with GATA4 mRNA expressed the pan-myocardial marker myl7 (Latinkic et al., 2003). To test if the cardiac tissue induced by GATA4 included diversification of different cell types, we first examined the expression of the ventricular marker myl3 at stage 39–40, when it becomes restricted to the developing ventricle within the heart (Smith et al., 2005). Whole mount in situ hybridisation (WMISH) analysis showed regional expression of myl3 in the majority of explants (Fig. 1A, C; 75%, n = 50). In addition, explants were analysed using double WMISH for expression of the cardiac troponin (tnni3) and myl3 markers (Fig. 1F, H). The heart of the control whole embryos at stage 39 showed an overlapping expression between tnni3 and myl3 in the ventricle region and tnni3 expression alone in the region morphologically corresponding to the atria (Fig 1I, J). We could not verify directly atrial cell fate identity since no specific markers are available in *Xenopus* for the stages of development that were focus of this study (see Introduction). GATA4-injected AC explants showed similar gene expression pattern to embryonic hearts, with areas of overlapping expression between tnni3 and myl3 (36%) and regions of tnni3 expression alone seen in 13% of explants (Fig. 1F, H; n = 55). Similar result has been found in GATA4 injected AC explants analysed using double-WMISH for the expression of myl3 and another pan-myocardial marker, myl7 (41% (n = 41) data not shown). Induction of ventricular cell fate in AC explants by GATA4 was also confirmed by the expression of another marker, irx4 (Fig. 1K; irx4 has prominent extracardiac expression and is expressed at a much lower level than myl3 (Garriock et al., 2001), making myl3 our marker of choice for ventricular cell fate). Together with the ventricular cell fate, we also found that GATA4 induced proepicardial cell fate differentiation by the expression of the proepicardial markers tbx18 and wt1 (Figs. 1K and 2C). Even though these markers are not epicardial-specific, the expression of both markers detected by RT-PCR in explants and the expression of tbx18 detected by WMISH in GATA4-injected cells in vivo (see below) is consistent with proepicardial cell fate. GATA4 was shown to be required for normal development of the proepicardium in the mouse embryo (Watt et al., 2004) and our finding that GATA4 is also sufficient to induce proepicardial cell fate in pluripotent AC explants confirms and extends this result.

### 3.2 GATA4-induced ventricular and proepicardial cell fates require tissue integrity

To gain insights into the mechanisms of cardiac cell diversification induced by GATA4 in AC explants we dissociated them and subsequently reaggregated (thus creating “reaggregates”). This procedure disrupts cell–cell contacts and any heterogeneity, or patterning that was established by this developmental stage, such as the anterior–posterior axis (Sokol and Melton, 1991). We found that GATA4 induced cardiac differentiation in reaggregates, as shown by expression of myl7, but the expression of the ventricular and proepicardial markers was lost (Fig. 2). This finding offered an opportunity to study the mechanisms involved in the establishment of cardiac cell type diversification. As ventricular tissue is located anteriorly within the heart, we have attempted to anteriorise reaggregates by using secreted Wnt antagonist, Dkk-1. Co-expression of GATA4 and Dkk-1 in reaggregates was sufficient to restore the expression of myl3, but not of proepicardial markers (Fig. 2B). When analysed by WMISH, 50% of GATA4 and Dkk-1 co-injected AC explants expressed myl3 (n = 25) (data not shown). We have initially considered the importance of spatially restricted delivery of Dkk-1 by conjugating Dkk-1 expressing AC explants with GATA4-injected reaggregates, and we observed the same result as with uniform expression of Dkk-1 (data not shown), suggesting that specification of ventricular fate does not require anterior–posterior gradient of Wnt signalling, and that instead anterior tissue character is sufficient. Additional anteriorising treatment, through inhibition of BMP signalling via dominant-negative BMP type I receptor (Graff et al., 1994), resulted in restoration of both ventricular and proepicardial gene expression in GATA4-injected reagreggates (Fig. 2A). Thus, in this model cardiac cell fate diversification is driven by GATA4 in a process that critically requires tissue integrity and anterior character. In addition, BMP pathway inhibition may have an instructive role in this process.

### 3.3 GATA4 injected reaggregates generate a secondary heart-like structure when transplanted into a host embryo

**Fig. 3:**
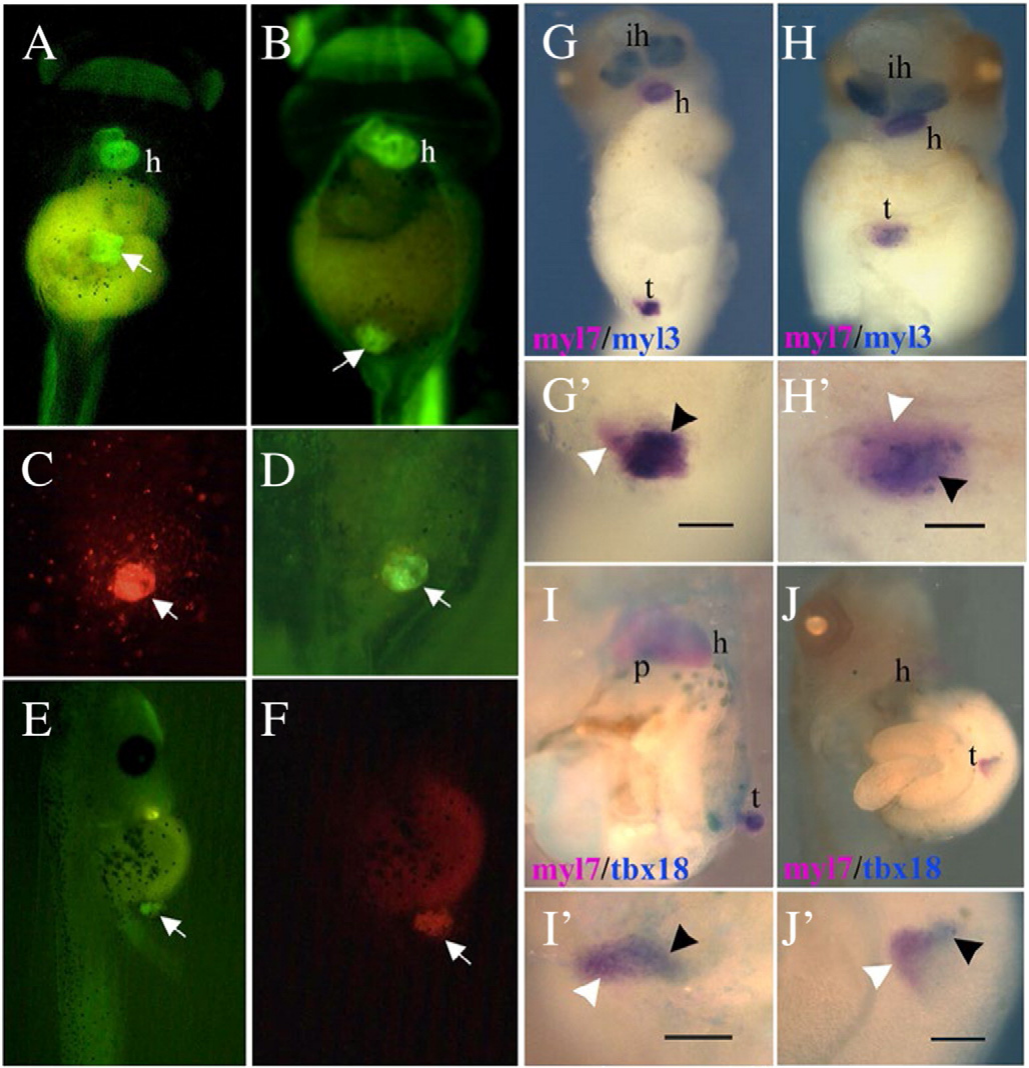
Ventricular and proepicardial gene expression is restored in reaggregated animal cap explants expressing GATA4 upon transplantation into host embryos. (A–F) Dissociated and reaggregated GATA4 injected explants (CAG-GFP) were transplanted at stage 20 into host (CAG-GFP) at the same stage. GFP expression was recorded at stage 39. (C, F) lineage tracing (rhodamine dextran) of transplants in D and E. (G, H) Transplants were analysed for the expression of myl7 and myl3. 48% of transplants (n = 45) showed patterned gene expression with regions of overlapping expression between myl7 and myl3 corresponding to the ventricle and regions expressing myl7 alone. 30% of transplants expressed only myl7, and in 17% there was a complete overlap between myl7 and myl3. (G′, H′) Close-up of transplants shown in G and H. Black head arrow shows overlapping gene expression and white head arrow shows region of myl7 expression alone. (I, J) 65% of transplants (n = 30) express both Tbx18 and myl7. (I′,J′) Close-up of transplants shown in I and J. h, heart; ih, interhyoid muscle of the jaw; p, proepicardium; v, ventricle. The scale bar corresponds to 100 μm.

We have shown that antagonism of Wnt and BMP signalling can restore aspects of cardiac cell diversification in GATA4-injected reaggregates. Another potential means of inducing a more complete process of cardiogenesis in GATA4 reaggregates is by transplantation into a host embryo, as reported by Ariizumi et al. for Activin-treated reaggregates [44]. In our modification of the assay, we used a cell-autonomous trigger of cardiogenesis, GATA4, with fewer additional effects (i.e., lower induced cellular complexity) compared to Activin. GATA4-injected reaggregates were transplanted at stage 20 in ventro-posterior region of sibling embryos at the same stage. To allow real time imaging of cardiogenesis of transplanted reaggregates (“transplants”) we used transplants derived from transgenic embryos expressing GFP in striated muscle under control of cardiac actin promoter, CAG-GFP [46]. Previous work has established that GATA4 induces cardiac but not skeletal muscle in animal cap explants (Latinkic et al., 2003), thus allowing us to unambiguously interpret GFP activity as a readout of cardiac differentiation. At stage 39–40 100% of transplants showed GFP activity and 46% (n = 220) were beating (Fig. 3A, F). In addition, double WMISH confirmed that the transplants expressed ventricular marker myl3 and proepicardial marker tbx18 when analysed at stage 40 (Fig. 3 — 65% of the reaggregates show patterned myl3 and tbx18 expression; n = 30). Cardiac tissue within transplants underwent rudimentary morphogenesis, as evidenced by the formation of tube-like structures (Fig. S1). Transplants, independently from the structure gained at stage 39–40, showed beating activity. Thus, transplantation of GATA4-injected reaggregates into host embryos results in formation of secondary heart-like (SH) structures, with patterned ventricular and proepicardial gene expression (Fig. 3), frequent beating and with aspects of cardiac morphogenesis.

### 3.4 Embryonic axes of the host embryo are required for the development of secondary heart-like structure

**Fig. 4:**
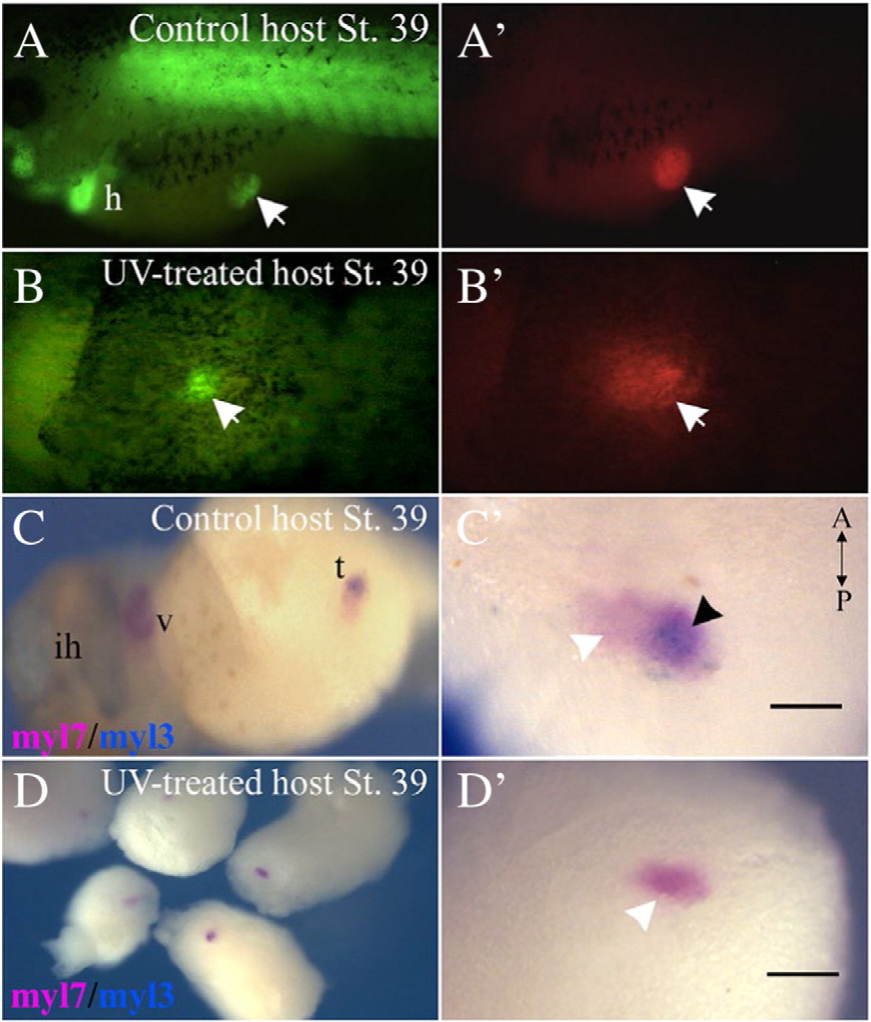
Secondary heart formation is lost in UV-ventralised hosts. (A, B) GATA4 injected reaggreagates (CAG-GFP) were transplanted at stage 20 in control (A, A′) or UV-treated hosts (B, B′) at the same stage. Transplants were viable (A′, B′) and were also positive for GFP expression in both control and UV-transplants analysed at stage 39. (C, D) Double-WMISH analysis for the expression of myl7 and myl3 indicated that whilst control transplants showed patterned expression of both myl7 and myl3 markers (C), transplants in UV-hosts (D) only expressed myl7 expression (white arrowhead). (C′, D′). Higher magnification of transplants shown in C and D. Region of overlap between myl3 and myl7 (purple) is indicated by black arrowhead. h, heart; ih, interhyoid muscle of the jaw; t, transplant; v, ventricle. The scale bar corresponds to 100 μm.

We showed that GATA4-injected reaggregates transplanted at stage 20 into isochronic host embryos developed a secondary heart-like structure (Fig. 3). To begin characterisation of the nature of the host-transplant interaction that results in the development of SH, we first assessed the role of axial patterning of the host. We transplanted GATA4 injected reaggregates into UV-ventralised host embryos with severe dorso-anterior truncations. Transplants present in UV-treated hosts showed evidence of cardiogenesis when analysed at stage 35 (CAG-GFP activity; Fig. 4A, B). This was confirmed using WMISH analysis for the expression of myl7, showing that cardiac induction triggered by GATA4 in AC explants was maintained in UV-ventralised hosts (Fig. 4D — 35% n = 65). More significantly, transplants placed into UV-treated hosts did not beat and also failed to express ventricular marker myl3 when analysed at stage 39 (Fig. 4D′). In addition, the size of heart tissue in transplants appeared to be significantly smaller in UV-treated hosts, an observation that was followed up below (Fig. 8D and Fig. S3). These results suggested that normal axial patterning of the host is required for the formation of the secondary heart and that the host does not merely provide trophic support. Closer analysis of experiments with control hosts (R20H20) showed that the axis defined by the relative position of ventricular, non-ventricular myocardial and proepicardial tissue within SHs (Fig. 3) did not show consistent correlation with embryonic axes of the host embryo (data not shown). Therefore, the ventricular and proepicardial cell fates are not specified by a mechanism that is directly related to the embryonic axes. Taken together, our results argue in favour of an essential permissive role of the primary embryonic axis in specification of ventricular and proepicardial cell fates.

### 3.5 Ventro-lateral embryonic areas permit secondary heart formation

**Fig. 5:**
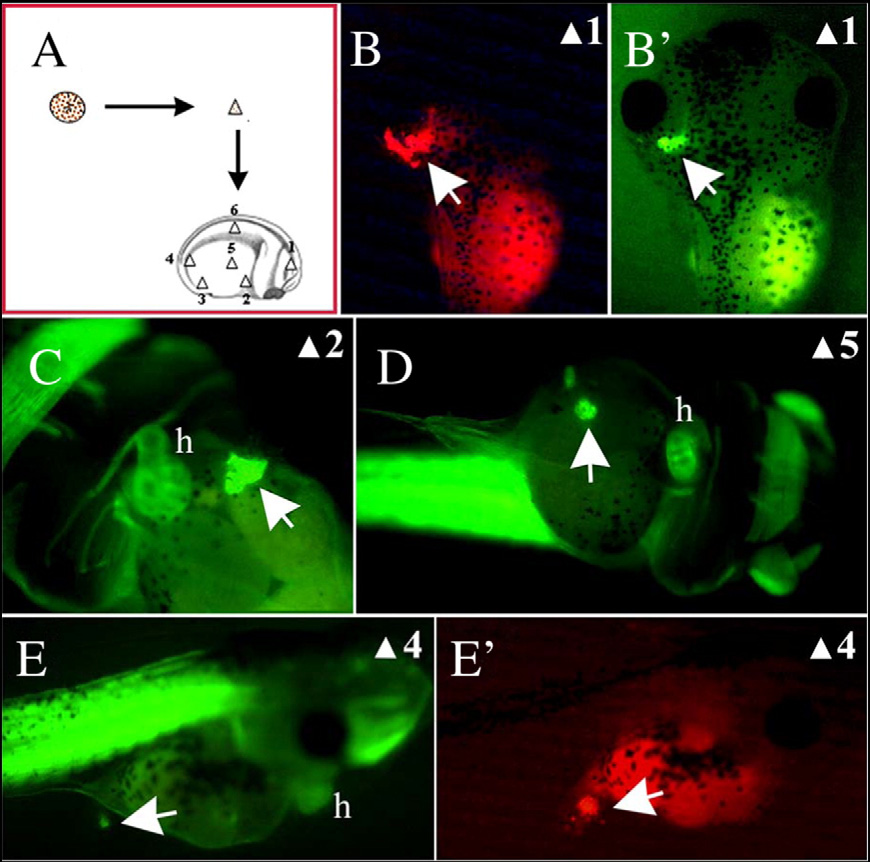
Host shows broad spatial competence for the development of the secondary heart. (A) GATA4 injected reaggregates (CAG-GFP) were transplanted at stage 20 into host embryos (CAG-GFP) at the same stage in a number of different regions, as indicated by the number. (B, B′) 51% of transplants in the head showed GFP expression but did not beat (n = 20). (C–E′) Transplants in the lateral (position 5) and the caudal side (position 4) of host embryos developed beating SH as control ventral transplants. Transplant were all analysed at stage 39. (B, E′) lineage tracing (rhodamine dextran) of transplants whose cardiac GFP activity is shown in B′ and E, respectively. h, heart.

**Fig. 6:**
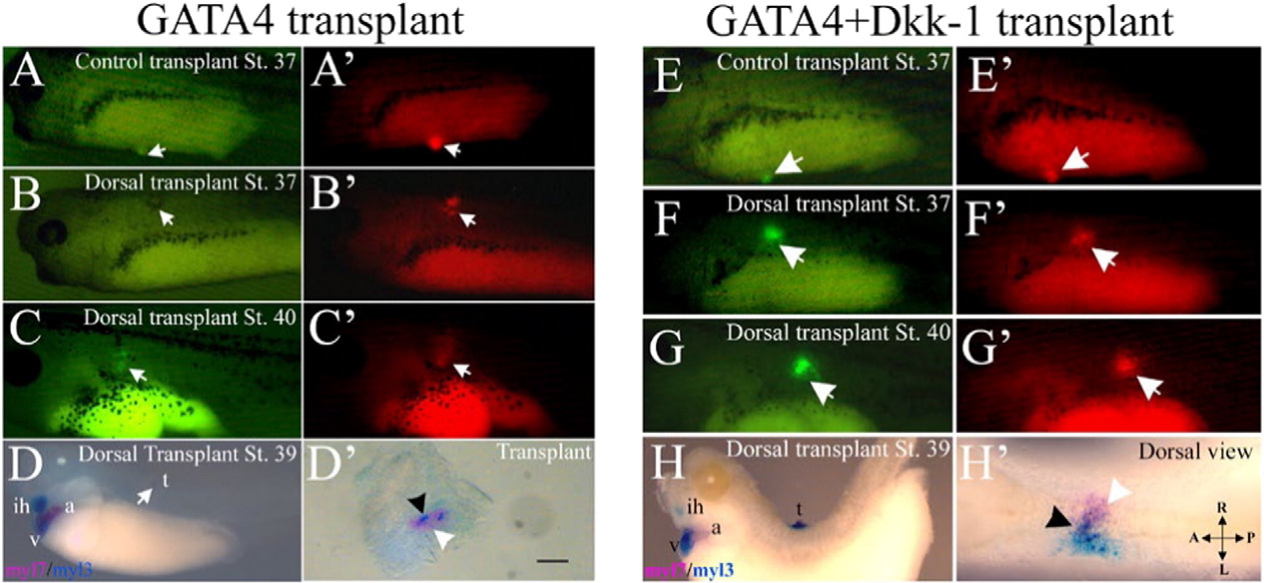
Dorsal GATA4-expressing transplants show a Dkk-1 sensitive delay of cardiogenesis. (A, A′) Control transplants of GATA4-injected reaggregates (CAG-GFP) showed cardiac differentiation by GFP expression at stage 37 as indicated by the arrows. (B, B′) In contrast, transplants placed in the dorsal side of the host (position 6 in Fig. 5A) were negative for GFP expression at stage 37 (100%; n = 20), but 50% were positive after stage 39 (C, C′). No beating activity was detected in these reaggregates. (D, D′) Double-WMISH showed that 20% (n = 94) of dorsal transplants express myl7 alone (white arrowhead) and only a small group of cells express both myl7 and myl3 (black arrowhead). (E–F′) Both control and dorsal transplants of reaggregates (CAG-GFP) co-injected with GATA4 and Dkk-1 showed evidence of cardiac differentiation (GFP activity) at stage 37. (G, G′) Cardiac expression is maintained in dorsal transplants at stage 40. Beating activity was never detected in dorsal transplants. (H, H′) Double WMISH shows overlapping expression between myl7 and myl3 (black arrowhead) and regions of myl7 expression alone (white arrowhead) in 35% of dorsal transplants (n = 84; the remaining transplants were negative for cardiac gene expression). a, atrium; ih, interhyoid muscle of the jaw; t, transplant; v, ventricle. The scale bar corresponds to 100 μm.

The ability to generate a SH from GATA4-injected reaggregates depends on the host embryo. To identify the region or regions in the host capable of supporting the development of a SH, we transplanted GATA4 injected reaggregates (CAG-GFP) at stage 20 in different locations of the host at the same stage (Fig. 5). Transplants placed in the head region of the hosts were not beating, despite being capable of cardiac differentiation (50%, n = 20, Fig. 5B). In contrast, transplants in the lateral and caudal region of the host were beating (Fig. 5C,E) similarly to control reaggregates transplanted in the ventral region of the host (Fig. 3). GATA4 injected reaggregates transplanted in the dorsal side of the host lacked cardiac gene expression when analysed at stage 37 (100%; n = 20) with respect to control transplants at the same stage (Fig. 6B). When the same set of transplants was analysed at stage 40 they showed cardiac gene expression (50% n = 20) but did not beat (Fig. 6C). Dorsal transplants were also analysed using double WMISH for the expression of myl7 and myl3 and a small region of overlapping expression between these markers was seen (Fig. 6D′) in contrast to expression pattern found in control reaggregates (Fig. 3). Therefore, dorsal transplants of GATA-4 induced cells showed delayed and less effective cardiac differentiation and cell fate diversification.

The delay in cardiomyocyte markers expression in reaggregates transplanted into the dorsal region of the host might have been caused by Wnt/β-catenin signalling, which is known to be high at the dorsal midline and to be a negative regulator of cardiogenesis (Schneider and Mercola, 2001, Tzahor and Lassar, 2001). To test this scenario we used reaggregates co-injected with mRNAs encoding GATA4 and Wnt antagonist Dkk-1 and transplanted them at stage 20 in the dorsal side of the host and in the ventral side of the host as control transplants (Fig. 6E–H′). When dorsal transplants were analysed at stage 37, consistent cardiac gene expression was found (90% n = 20) as indicated by the expression of GFP (Fig. 6F, F′). These transplants were also analysed by double WMISH at stage 40 for the expression of myl7 and myl3 (Fig. 6H, H′). The results showed a region of overlapping expression between myl7 and myl3 and a region positive for myl7 only (Fig. 6H, H′), similar to the one seen in control transplants. Despite showing robust and timely cardiac differentiation, dorsal transplants injected with GATA4 and Dkk-1 did not beat, similar to GATA4-injected reaggregates transplanted in the head and in the dorsal region of the host embryos. These embryonic regions apparently restrict the development of physiological maturation of the myocardium.

### 3.6 Secondary heart can be formed until just prior to cardiac differentiation

**Fig. 7:**
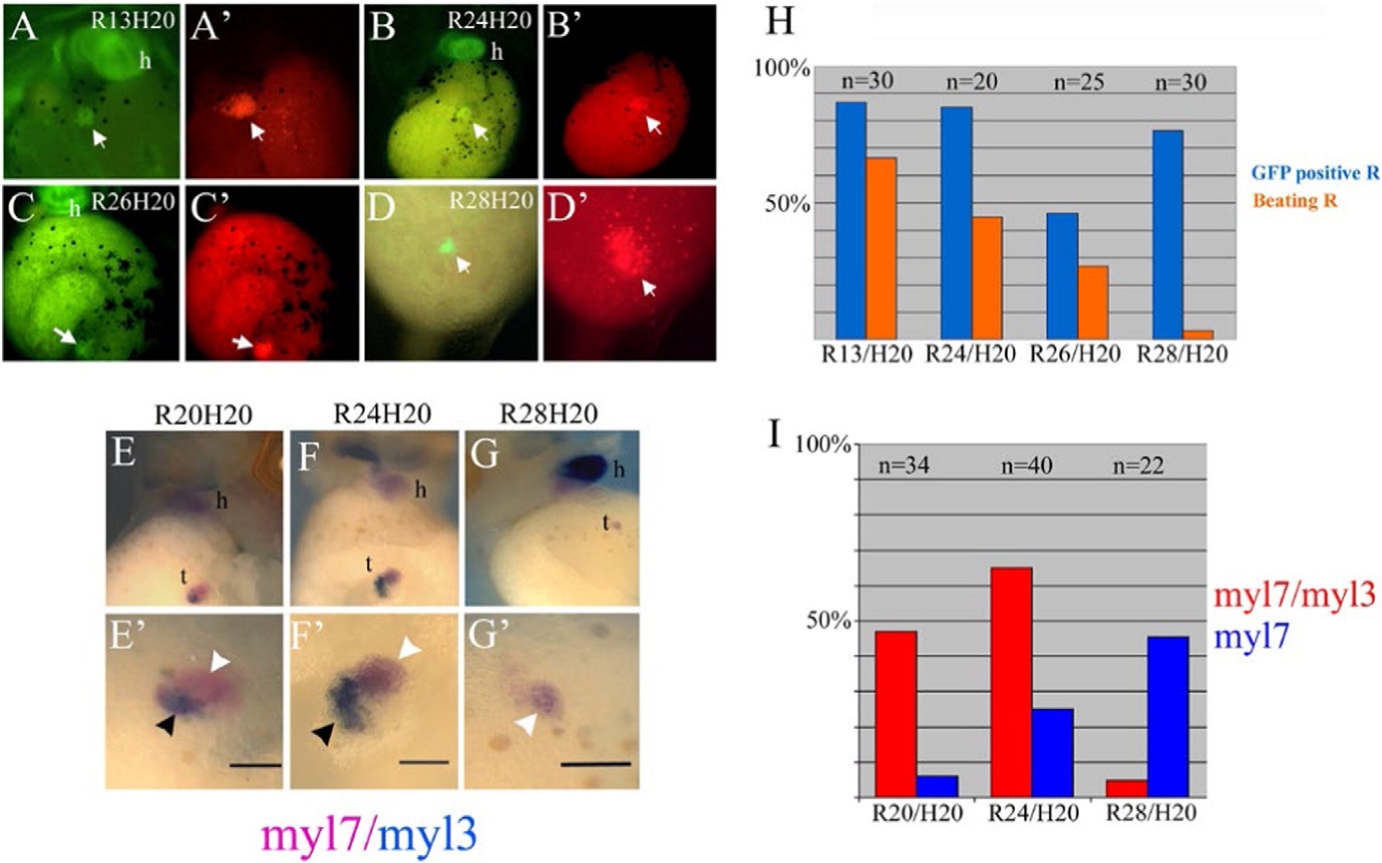
Competence to generate a secondary heart is maintained until the onset of cardiac differentiation. GATA4-injected animal cap reaggregates (CAG-GFP) were transplanted at stage 13 (A, A′), 24 (B, B′), 26 (C, C′) and 28 (D, D′) in the ventral side of host embryos at stage 20 (CAG-GFP) and were analysed at stage 39–40 for cardiac GFP expression and for lineage tracing (rhodamine-dextran). (E–G′) Patterned expression of myl7 and myl3 was found in reaggregates transplanted at stage 20 and 24. Overlapping expression is indicated by black arrowheads and myl7-only expression by white arrowheads. (H) Summary of frequency of GFP expression and beating activity. The capacity to generate a beating structure declines with the increasing age of the transplants. (I) Data summarised the number of embryos positive to the gene expression of myl7 and myl3 in double WMISH. h, heart; t, transplant. The scale bar corresponds to 100 μm.

**Fig. 8:**
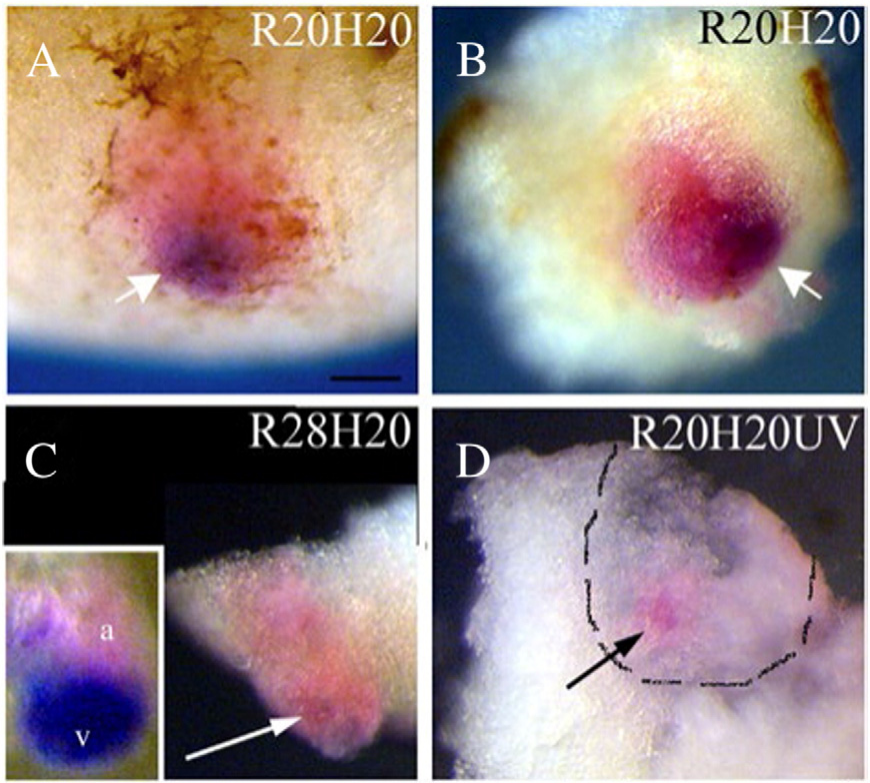
Growth of secondary hearts is dependent on the host embryo. Transplants in samples analysed by double-WMISH for myl7 and myl3 at stage 39 were visualised by lineage tracing (pink-light red colour). (A, B) Examples of control transplants (R20H20; GATA-4 injected reaggregates transplanted at stage 20 into host embryos at the same stage) show that cardiac tissue occupies a substantial part of the transplant. In contrast, cardiac tissue in (C) R28H20 and (D) R20H20UV (UV-ventralised hosts) transplants occupies a much smaller area (arrows). (D) Inside view of a dissected sample is presented to better show cardiac tissue (myl7, pointed by an arrow). Lineage trace that is visible from the outside is outlined. The scale bar corresponds to 100 μm. All images except the insert in (C) (showing the heart dissected from control embryo at stage 39) are at the same magnification. At least 5 samples were analysed for each treatment, with comparable results.

To define the time window within which GATA-4 injected reaggregates can generate a SH, we performed a series of transplantation experiments using reaggregates of different developmental stages at the time of transplantation and host embryos at stage 20 (Fig. 7). Analysis of these transplants showed that all developed SHs that were capable of beating, with the exception of those transplanted at stage 28 (Fig. 7A, D). In addition, stage 28 transplants had a different morphology when compared to younger transplants, as they were not compact but instead contained loosely arranged cells, some of which were migrating into the host embryo (Fig. 7D, D′). In contrast to reaggregates transplanted at stage 20 and 24, which showed expression of both myl7 and myl3 markers (Fig. 7E, F), reaggregates transplanted at stage 28 showed the expression of myl7 only (Fig. 8G). Thus, the competence of GATA4-expressing transplants to generate a secondary heart in which cardiac cell diversification has occurred exists before stage 20 and gradually declines until it is completely lost by stage 28 (Fig. 7). Stage 28 transplants behaved like GATA4 injected reaggregates cultured in vitro. These results suggest that cardiac precursors can receive the signals required for SH formation within a relatively broad time window.

To test whether our findings obtained with GATA4-expressing animal cap explants might be applicable to native cardiac precursors, we investigated ventricular and proepicardial cell fate specification in heart field (HF) explants. The HFs are able to grow and differentiate autonomously in culture and have the capacity to generate a looped beating structure (Raffin et al., 2000). WMISH analysis for the expression of myl7 and myl3 showed a region of overlapping expression of myl7 and myl3 as well as a region expressing myl7 alone (Fig. S2) similarly to what was found in control embryos and control transplants at the same stage. RT-PCR analysis of HFs at stage 40 confirmed the expression of irx4 and tbx18. In contrast, heart field explants that were subjected to dissociation/reaggregation expressed pan-myocardial marker myl7 only, suggesting that tissue integrity is essential for ventricular and proepicardial gene expression similarly to GATA4-induced reaggregates (Fig. S2). Dissociation of HF explants reproducibly led to a decrease in cardiac differentiation (Fig. S2), suggesting that tissue integrity is important for the maintenance of specified cardiac fate. This is in contrast with dissociated AC explants expressing exogenous cell-autonomous cell fate specifier GATA4, which presumably makes cardiogenesis under those conditions less sensitive to tissue disruption. Taken together, our findings suggest that in embryos ventricular and proepicardial cell fates are not stably specified at stage 20, many hours after cardiac induction and that this aspect of cardiogenesis is reproduced in a process regulated by GATA4 in AC explants.

### 3.7 Growth of secondary hearts

Secondary hearts showed an enhancement in cardiogenesis that is clearly lost in transplants at stage 28 and in transplants placed in UV-treated hosts. We documented the extent of cardiac differentiation (relative size of cardiac area) in secondary hearts (R20/H20) compared to R20/H20UV and R28/H20 transplants. Real-time analysis was performed using CAG-GFP transplants in both UV treated and wild-type hosts at stage 39. The CAG-GFP positive area within rhodamine–dextran labelled transplants was assessed by image analysis, which showed that R20/H20-UV transplants consistently have smaller CAG-GFP positive (cardiac) area when compared with R20/H20 control transplants (Fig. S3). This conclusion was confirmed when embryos were analysed using double-WMISH for the expression of myl7/myl3 (Fig. 8). Lineage tracing confirmed that the transplant size was comparable in all three treatment groups, however, the area occupied by cardiac tissue within the transplants was greater in R20/H20 samples compared to R20/H20UV and R28/H20 (Fig. 8). Three-dimensional shape and variable location of transplants made it difficult to quantify this effect and have limited our analysis to qualitative assessment. Nonetheless, our observations suggest that cardiac tissue expands in SHs and that the growth may be regulated in a similar manner as cardiac cell diversification in the heart. The loss of competence of transplanted stage 28 cardiac precursors to respond to the signals from the host, and the disruption of those signals in UV-treated hosts, prevent growth of cardiac tissue in transplants and the formation of SH.

## 4 Discussion

In this study we have developed an experimental model to investigate cell fate diversification during vertebrate heart development. This model is based on pluripotent animal pole cells from *Xenopus* embryos in which cardiogenesis was triggered by the expression of GATA4. Whereas induction of expression of pan-myocardial markers is a cell-autonomous process, specification of ventricular and proepicardial fates by GATA4 requires cell–cell communication and can be restored in reaggregated explants by inhibition of BMP signalling. Cardiac cell diversification can also be restored in GATA4-expressing animal pole cells by transplantation into a host embryo. Transplants in addition generated a secondary heart-like structure that developed rhythmical beating, underwent rudimentary morphogenesis and grew, all features of heart development that do not commonly occur in cultured animal cap explants in vitro injected with GATA4.

### 4.1 Inhibition of BMP is sufficient to specify ventricular and proepicardial (VP) fates during GATA4-induced cardiogenesis

Loss of VP cell fates caused by dissociation of GATA4-expressing explants implies that the required factor was either secreted and present at the time of dissociation, or that its production requires specific spatial arrangement of cells in the explant that is lost by dissociation. Our evidence suggested that an extracellular gradient is not required, since uniform expression of a cell-autonomous reagent, dominant-negative BMP receptor I, is capable of restoring VP cell fates. Inhibition of BMP signalling leads to anteriorisation of animal pole explants and it is possible that this plays a role in inducing ventricular cell fate, which is anterior within the heart, in GATA4-expressing reaggregates. Consistent with the view that BMP inhibition acts to anteriorise cardiac tissue, anteriorisation via Wnt antagonist Dkk-1 can rescue ventricular specification in GATA4-expressing reaggregates. However, our data shows that BMP inhibition rescues proepicardial cell fate as well, arguing that BMP signalling has an additional instructive role in cardiac cell fate diversification.

Animal pole ectodermal explants are known to contain BMP ligands, and their removal by dissociation (Godsave and Slack, 1989, Grunz and Tacke, 1989) or blocking by secreted antagonist Noggin (Lamb et al., 1993) induces neural cell fate. Here we have shown that expression of GATA4 in intact animal caps induces panmyocardial, ventricular and proepicardial cell fates, suggesting that endogenous BMP is at least permissive for the process. We have also shown that inhibition of BMP signalling via truncated BMP receptor can restore VP marker expression in dissociated GATA4-expressing animal cap explants. This apparently paradoxical result indicates that perhaps endogenous BMP and/or tissue integrity are required to generate a BMP antagonist which acts at a later stage, or that the effect is quantitative (requirement for a greater level of BMP inhibition than can be afforded by AC dispersal).

Our findings are in agreement with studies on zebrafish embryos that have shown that inhibition of the BMP pathway is required in the cardiac field and that it favours ventricular cell fate specification (de Pater et al., 2012, Marques and Yelon, 2009).

### 4.2 Broad ventro-lateral areas of a host embryo support secondary heart development

The secondary heart formation assay we used in the current study is a modification of the assay described by Ariizumi and colleagues, in which dissociated and reaggregated cells were treated with Activin and transplanted into host embryo at stage 20 (Ariizumi et al., 2003). Activin is a well-known mesoderm (Asashima et al., 1990, Green et al., 1990, Smith et al., 1990) and cardiac tissue inducer (Logan and Mohun, 1993). As GATA4 acts downstream of Activin/nodal signalling we used it as a simpler model for cardiogenesis. We found that GATA4-injected reaggregates transplanted into host embryos displayed beating and expression of ventricular tissue (myl3^+^/myl7^+^) adjacent to a presumptive atrial compartment (myl3^−^/myl7^+^; Fig. 3). The SH also expressed proepicardial marker tbx18 demonstrating that a further heart cell diversification has occurred (Fig. 3). In addition, we documented growth (Fig. 8) and aspects of cardiac morphogenesis (Fig. S1) of SHs. All these attributes of SHs critically depend on the host embryo, which provides broad permissive regions, ventrolateral and caudal, where the beating SH can develop (Fig. 5). There is no obvious cellular contribution from the host to the SH (Fig. S4). Thus permissive regions of the host contribute a signal, or signals, which direct SH formation from transplanted cardiac precursors.

In contrast, head and dorsal midline did not support SH formation. Dorsal transplants showed a delay in expression of cardiac markers and did not beat (Fig. 6). Dorsal midline region is regulated by the expression of Wnt signals which have been shown to repress cardiogenesis in the chick neural tube (Tzahor and Lassar, 2001). In *Xenopus*, activation of Wnt/β-catenin pathway during gastrulation has no effect on cardiogenesis, while the same pathway blocks cardiogenesis if activated after cardiac specification and before the onset of cardiac differentiation (Samuel and Latinkic, 2009). Our results provide evidence that the delay of cardiac differentiation in dorsal transplants is mediated by Wnt/β-catenin pathway, as it is relieved by coexpression of GATA4 and Wnt antagonist Dkk-1. GATA4 and Dkk-1 co-injected transplants showed cardiac gene expression from stage 37 as in control transplants (Fig. 6). In these experiments Dkk-1 is presumably acting by protecting the transplants from the inhibitory activity of Wnt pathway.

### 4.3 Embryonic axes of the host are required for secondary heart development

The host does not merely provide trophic signals for the development of cardiac precursors present in the transplants. Transplants placed in UV-treated host express pan-myocardial marker myl7 but not ventricular marker myl3 (Fig. 4D) and do not grow (Fig. 8). Thus, transplants placed into UV-treated hosts behave as if they developed in vitro, in culture (Fig. 2A). How could embryonic axes be involved in SH formation? Since broad range of embryonic regions supports development of SH, we believe that it is unlikely that axial structures are directly involved. The host most likely provides a signal or signals that are permissive for the formation of the SH. Retinoids are known to regulate multiple aspects of anterio-posterior patterning, including heart development (Xavier-Neto et al., 2015), and could be involved in mediating the permissive signal that we have identified. Of interest, inhibition of retinoic acid signalling, together with BMP pathway inhibition by Noggin, promotes development of ventricular cardiomyocytes from differentiating human Embryonic Stem cells, whereas BMP inhibition together with retinoic acid treatment encourages atrial myocyte development (Zhang et al., 2011). In our work we were limited by the lack of an atrial-specific marker, and it is possible that cardiac tissue observed in transplants placed in UV-treated hosts represented atrial, rather than pan-myocardial fate. This scenario would be consistent with higher level of retinoic acid signalling in posteriorised UV-treated embryos.

### 4.4 Capacity to generate a secondary heart is lost by the onset of cardiac differentiation

The capacity of GATA4-expressing transplants to develop a SH is extended after the end of gastrulation to the post-neurula stages (Fig. 7). However, it decreases at the beginning of the cardiac differentiation and it is lost just before the linear heart tube stage (stage 28; Fig. 7). The reaggregates transplanted at stage 28 did not beat when analysed at stage 40 (Fig. 4). Instead of a compact structure that forms from control (stage 20) transplants, stage 28 transplants were looser with evidence of extensive cell migration into the host embryo (Fig. 4). The loss of capacity to generate a SH is likely to be an intrinsic property of stage 28 GATA4-expressing transplants, and is reminiscent of heart field restriction, in which heart field cells that are competent to form the heart lose this capacity at stage 28 (Sater and Jacobson, 1990). The relatively broad time window within which SH can be specified implies plasticity of cardiac precursors to the signal(s) which must be acting during that period.

### 4.5 *Xenopus* explants as a model to study heart organogenesis

The current challenge in directed cardiac differentiation is to produce relatively pure populations of defined and mature cardiac cells, as commonly used protocols usually yield a non-specific mixture of immature cardiomyocytes (Burridge et al., 2012, Vidarsson et al., 2010). Here we have shown that ventricular and proepicardial cell fates can be specified by inhibition of BMP signalling from panmyocardial-fated precursors, and that cardiac precursors retain plasticity to be induced to organise a secondary heart until the onset of cardiac differentiation. Together with prior work that has shown self-assembly of a functional secondary heart from induced animal pole ectodermal cells (Ariizumi et al., 2003, Kinoshita et al., 2010), these studies establish *Xenopus* embryo as a model to investigate cardiac cell diversification and organogenesis, that complements existing models.

The following are the supplementary data related to this article.

Fig. S1 Morphogenesis of second hearts. Confocal microscopy was performed on SH (n = 41; 73% beating) which were visualised by immunohistochemistry using anti-tropomyosin antibody CH1. (A) 35% of the SH showed morphology similar to a linear heart tube whilst (B) remaining SH were unstructured. (B) shows skeletal muscle staining in addition to SH.

Fig. S2 Cardiac cell fate diversification requires intact heart fields. (A) Diagram of the experiment. Heart Fields (HF) were explanted at stage 20 and were analysed at stage 40. (B) Dissociated and reaggregated HFs do not express ventricular and proepicardial markers, which are expressed by intact HF explants as well as by HFs from which endoderm was removed. RT-PCR analysis was performed for indicated markers. Double-WMISH analysis showing regions of overlapping expression between myl7 and myl3 in (C, D) control sibling embryos at stage 39 (C — lateral view, D — ventral view) and in HF explants (E, F). (E) All HF analysed express myl7 and myl3. (F) close up of 3 HF explants showing areas corresponding to the ventricle (myl7 +/myl3 +; black arrowheads) and myl7 +/myl3- (white arrowheads). a — atria, ih — interhyoid muscles of the jaw, v — ventricle, HF R; heart field dissociated and reaggregated, WE; whole embryos.

Fig. S3 UV-treated host does not support the growth of the reaggregate. Representative examples of (A) control transplants (n = 40) and (B) UV-treated transplants (n = 40) that were analysed at stage 39 using the fluorescent microscope. Transplants (CAG-GFP), marked with white rectangular, were positive in all transplant samples for GFP expression. The 2D analysis showed that 80% (n = 32) of transplants placed in UV-treated host embryos have smaller size when compared to control transplants. (A′, B′) Higher magnification of transplants into control and UV-treated hosts. The bar corresponds to 100 μm.

Fig. S4 Secondary heart develops without obvious cellular contribution from the host. GATA4-injected reaggregates (CAG-GFP) were transplanted at stage 20 into host embryos at the same stage. For this analysis GATA-4 was injected without lineage tracer (rhodamine dextran) and host embryos were injected with lineage tracer to facilitate visualisation of labelled host cells localisation. An area of a representative embryo carrying a transplant in the caudal area (arrows) shown to reveal (A) lineage tracer or (B) GFP-expressing secondary beating heart in the caudal region of the host. This low-resolution analysis offers no evidence of extensive cellular contribution of the host to the transplant; given the limitations of the method, a small number of host cell contribution to the transplant cannot be excluded. 20 beating transplants were examined with no evidence of host contribution in any.
