# Supplementary material for: Ventricular cell fate can be specified until the onset of myocardial differentiation
Source: Mech Dev. 2016 Feb;139:31–41. doi: 10.1016/j.mod.2016.01.001 (PMC4798847; doi:10.1016/j.mod.2016.01.001)
Supplement: Fig. S1 — Morphogenesis of second hearts. Confocal microscopy was performed on SH (n = 41; 73% beating) which were visualised by immunohistochemistry using anti-tropomyosin antibody CH1. (A) 35% of the SH showed morphology similar to a linear heart tube whilst (B) remaining SH were unstructured. (B) shows skeletal muscle staining in addition to SH. [file mmc1.pdf]

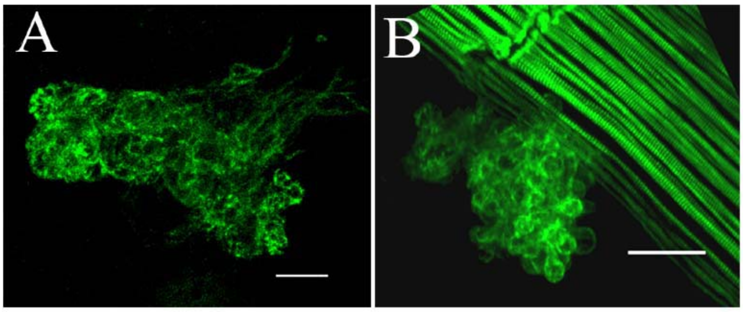

Figure S1

Figure S1. Morphogenesis of Second Hearts. Confocal microscopy was performed on SH (n=41; 73% beating) which were visualised by immunohistochemistry using anti-tropomyosin antibody CH1. (A) 35% of the SH showed morphology similar to a linear heart tube whilst (B) remaining SH were unstructured. (B) shows skeletal muscle staining in addition to SH.
