# Supplementary material for: Ventricular cell fate can be specified until the onset of myocardial differentiation
Source: Mech Dev. 2016 Feb;139:31–41. doi: 10.1016/j.mod.2016.01.001 (PMC4798847; doi:10.1016/j.mod.2016.01.001)
Supplement: Fig. S2 — Cardiac cell fate diversification requires intact heart fields. (A) Diagram of the experiment. Heart Fields (HF) were explanted at stage 20 and were analysed at stage 40. (B) Dissociated and reaggregated HFs do not express ventricular and proepicardial markers, which are expressed by intact HF explants as well as by HFs from which endoderm was removed. RT-PCR analysis was performed for indicated markers. Double-WMISH analysis showing regions of overlapping expression between myl7 and myl3 in (C, D) control sibling embryos at stage 39 (C — lateral view, D — ventral view) and in HF explants (E, F). (E) All HF analysed express myl7 and myl3. (F) close up of 3 HF explants showing areas corresponding to the ventricle (myl7 +/myl3 +; black arrowheads) and myl7 +/myl3- (white arrowheads). a — atria, ih — interhyoid muscles of the jaw, v — ventricle, HF R; heart field dissociated and reaggregated, WE; whole embryos. [file mmc2.pdf]

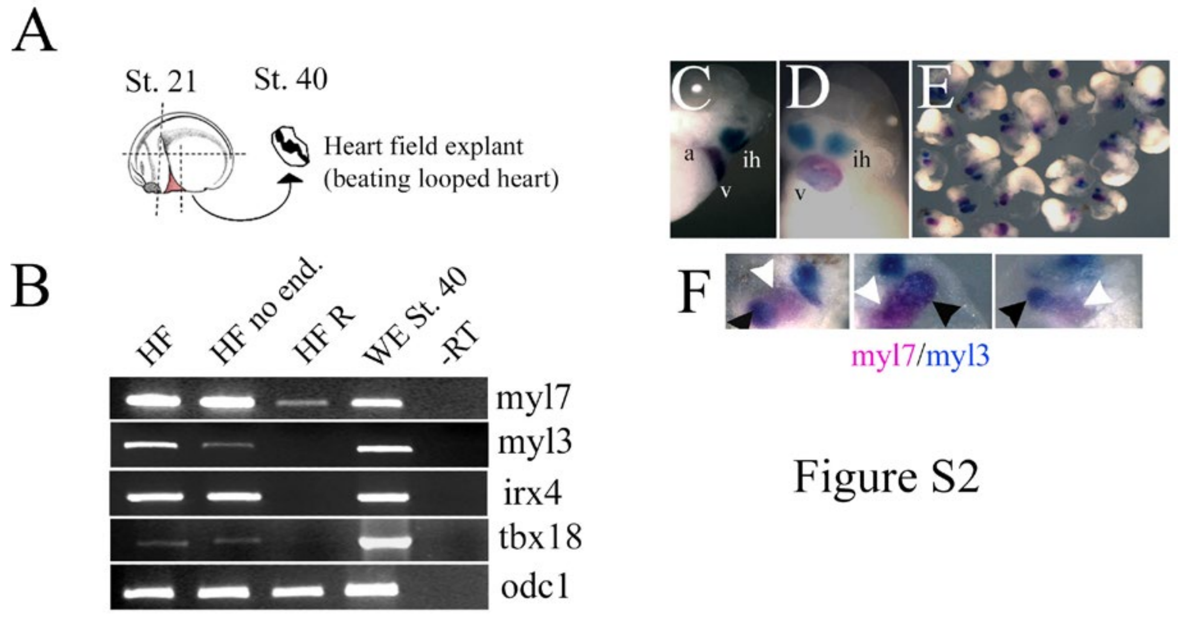

Figure S2

Figure S2. Cardiac cell fate diversification requires intact heart fields. (A) Diagram of the experiment. Heart Fields (HF) were explanted at stage 20 and were analysed at stage 40. (B) Dissociated and reaggregated HFs do not express ventricular and proepicardial markers, which are expressed by intact HF explants as well as by HFs from which endoderm was removed. RT-PCR analysis was performed for indicated markers. Double-WMISH analysis showing regions of overlapping expression between myl7 and myl3 in (C, D) control sibling embryos at stage 39 (C- lateral view, D- ventral view) and in HF explants (E,F). (E) All HF analysed express myl7 and myl3. (F) close up of 3 HF explants showing areas corresponding to the ventricle ($myl7^+$/$myl3^+$; black arrowheads) and $myl7^+$/$myl3^-$ (white arrowheads). a- atria, ih- interhyoid muscles of the jaw, v- ventricle, HF R; heart field dissociated and reaggregated, WE; whole embryos.
