# Supplementary material for: Ventricular cell fate can be specified until the onset of myocardial differentiation
Source: Mech Dev. 2016 Feb;139:31–41. doi: 10.1016/j.mod.2016.01.001 (PMC4798847; doi:10.1016/j.mod.2016.01.001)
Supplement: Fig. S3 — UV-treated host does not support the growth of the reaggregate. Representative examples of (A) control transplants (n = 40) and (B) UV-treated transplants (n = 40) that were analysed at stage 39 using the fluorescent microscope. Transplants (CAG-GFP), marked with white rectangular, were positive in all transplant samples for GFP expression. The 2D analysis showed that 80% (n = 32) of transplants placed in UV-treated host embryos have smaller size when compared to control transplants. (A′, B′) Higher magnification of transplants into control and UV-treated hosts. The bar corresponds to 100 μm. [file mmc3.pdf]

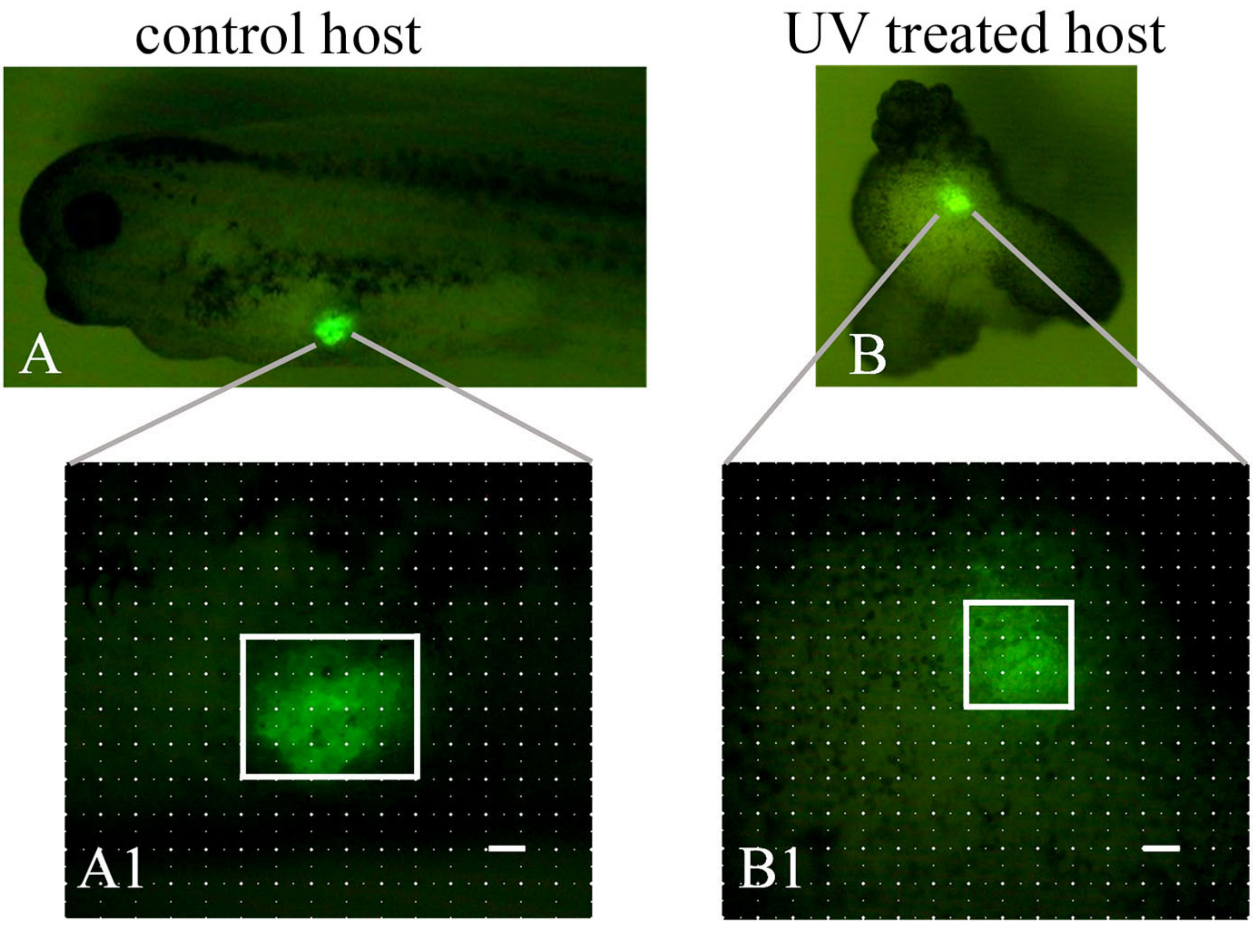

Figure S3

Figure S3. UV-treated host does not support the growth of the reaggregate. Representative examples of (A) control transplants (n=40) and (B) UV-treated transplants (n=40) that were analysed at stage 39 using the fluorescent microscope. Transplants (CAG-GFP), marked with white rectangular, were positive in all transplant samples for GFP expression. The 2D analysis showed that 80% (n=32) of transplants placed in UV-treated host embryos have smaller size when compared to control transplants. (A’, B’) Higher magnification of transplants into control and UV-treated hosts. The bar corresponds to 100µm.
