# Supplementary material for: Ventricular cell fate can be specified until the onset of myocardial differentiation
Source: Mech Dev. 2016 Feb;139:31–41. doi: 10.1016/j.mod.2016.01.001 (PMC4798847; doi:10.1016/j.mod.2016.01.001)
Supplement: Fig. S4 — Secondary heart develops without obvious cellular contribution from the host. GATA4-injected reaggregates (CAG-GFP) were transplanted at stage 20 into host embryos at the same stage. For this analysis GATA-4 was injected without lineage tracer (rhodamine dextran) and host embryos were injected with lineage tracer to facilitate visualisation of labelled host cells localisation. An area of a representative embryo carrying a transplant in the caudal area (arrows) shown to reveal (A) lineage tracer or (B) GFP-expressing secondary beating heart in the caudal region of the host. This low-resolution analysis offers no evidence of extensive cellular contribution of the host to the transplant; given the limitations of the method, a small number of host cell contribution to the transplant cannot be excluded. 20 beating transplants were examined with no evidence of host contribution in any. [file mmc4.pdf]

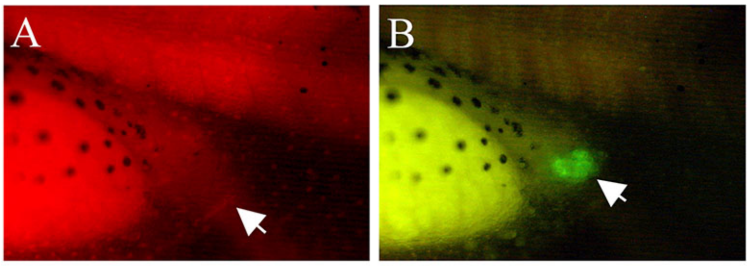

Figure S4

Figure S4. Secondary heart develops without obvious cellular contribution from the host. GATA4-injected reaggregates (CAG-GFP) were transplanted at stage 20 into host embryos at the same stage. For this analysis GATA-4 was injected without lineage tracer (rhodamine dextran) and host embryos were injected with lineage tracer to facilitate visualisation of labelled host cells localisation. An area of a representative embryo carrying a transplant in the caudal area (arrows) shown to reveal (A) lineage tracer or (B) GFP-expressing secondary beating heart in the caudal region of the host. This low-resolution analysis offers no evidence of extensive cellular contribution of the host to the transplant; given the limitations of the method, a small number of host cell contribution to the transplant cannot be excluded. 20 beating transplants were examined with no evidence of host contribution in any.
